# Chronic Diseases & Employment: An Overview of Existing Training Tools for Employers

**DOI:** 10.3390/ijerph16050718

**Published:** 2019-02-28

**Authors:** Fabiola Silvaggi, Matilde Leonardi, Erika Guastafierro, Rui Quintas, Claudia Toppo, Jerome Foucaud, Kristopher Lamore, Ulrike Rothe, Chiara Scaratti

**Affiliations:** 1 Neurology, Public Health, Disability Unit, Neurological Institute C. Besta IRCCS Foundation, 20133 Milan, Italy; matilde.leonardi@istituto-besta.it (M.L.); erika.guastafierro@istituto-besta.it (E.G.); rui.quintas@istituto-besta.it (R.Q.); c.toppo@campus.unimib.it (C.T.); chiara.scaratti@istituto-besta.it (C.S.); 2 Institut National du Cancer (INCa), 92100 Boulogne-Billancourt, France; jfoucaud@institutcancer.fr (J.F.); kristopher.lamore@gmail.com (K.L.); 3 Health Education and Practices Laboratory (LEPS EA 3412), Paris 13 University—UFR SMBH—74 rue Marcel Cachin, 93017 Bobigny CEDEX, France; 4 Laboratory of Psychopathology and Health Processes (EA 4057), Paris Descartes University, 92100 Boulogne-Billancourt, France; 5 Faculty of Medicine “Carl Gustav Carus”—Technische Universität Dresden, 01307 Dresden, Germany; ulrike.rothe@tu-dresden.de

**Keywords:** training tools, employment, chronic diseases, return to work, inclusion, employers, work ability

## Abstract

*Background*: The number of people living with one or more chronic diseases (e.g., neurological, musculoskeletal, cardiovascular, respiratory, metabolic disorders) has dramatically increased in recent decades, affecting all sectors, including the social and economic aspects of the work sector. In the frame of the European Union (EU) Joint Action “Chrodis Plus: Implementing good practices for chronic diseases”, a review has been performed in order to identify and analyze existing training tools for employers, including managers and Human Resources Staff (HRs), which aimed at creating and fostering inclusive and supportive workplaces for workers with chronic conditions and to avoid absenteeism, presenteeism, and early retirement. *Methods*: The training tools were identified through a revision of online published materials through Google Scholar and internet searches, published since 2006, in English, Italian, and Spanish. *Results*: The mapping of existing training tools highlighted the existence of two types of training tools: the first type includes those implemented by Social and Institutional Organizations (e.g., Patients’ Associations, Ministries, Unions), external to the company; the second involves those implemented by Large Multinational Enterprises. *Conclusions*: to promote an effective and concrete inclusion and participation of employees that are affected by chronic diseases in the labor market is necessary to involve employers and managers in training programs.

## 1. Introduction

The World Health Organization (WHO) broadly defines chronic or non-communicable diseases (NCDs) as diseases of long duration and generally slow progression, and are the result of a combination of genetic, physiological, environmental, and behavioral factors [1]. The prevalence of chronic diseases (that include e.g., neurological, musculoskeletal, cardiovascular, respiratory, and metabolic disorders) has been rising in Europe over the past decades, together with increasing the ageing of a population. It is estimated that one in four people of working age (15 to 64) lives with one or more long standing health problems that restrict their daily activities [2].

The level of income, the employment opportunities, the career, the social inclusion, and the working conditions of people with chronic diseases (PwCDs) are also affected. The comparative analysis on chronic diseases that was performed by European Commission [3] reports that the employment rate of PwCDs is less than half when compared to the economically active population, while the unemployment rate is twice. This analysis highlighted the difficulties and the barriers that were encountered by PwCDs in job acquisition and job tenure, and the related difficulties of the work sector in managing the presence of PwCDs.

Since 2009, OECD, in fact, reports a constant increase of NCDs in the work sector derived from the percentage of people requesting sick leave, taking early retirement and living on long term disability allowances, accounting about 10% of the labor force in some countries [4]. At the same time, OECD states that, due to the considerable ageing of the labor force, the labor market participation of PwCDs is necessary in order to cope with the reduction in labor supply, the shortage of skilled workforce, and the pressure on the pension system [5].

PwCDs face objective difficulties at the time of entering and re-entering in the labor market, which are often associated with psychological tension and uncertainty that frequently lead them to abandon the choice of return to work (RTW) after the onset of illness.

Moreover, prejudices and stereotypes that are related to certain chronic diseases still exist in the work sector and rigid forms of work organization penalize PwCDs [6]. Due to stigma and discriminatory attitudes from certain employers or colleagues, a lot of people with NCDs face difficulties in disclosing their condition and experience lack of support, as well as difficulties in obtaining new training opportunities and promotion, when they return to work after a sick leave or after a long-term absence [7]. Indeed, employers are often misinformed regarding chronic diseases and the abilities of people with NCDs to continue working. Raising awareness and information regarding chronic diseases and their impact on employment, as well as about the impact of employment on PwCDs, is therefore crucial, as also highlighted in the Final Recommendations [8] of the EU PATHWAYS Project (Participation To Healthy Workplaces And Inclusive Strategies in the work sector) (www.path-ways.eu).

The EU-project PATHWAYS carried out several actions to support a more inclusive employment sector, including a comprehensive systematic review to evaluate the effectiveness of existing strategies that target the integration and re-integration into work for persons with NCDs in Europe [9], highlighting the urgent need to improve and strengthen research on this field. PATHWAYS results showed that, in order to promote an inclusive labor markets for all, is crucial to redesign the role of the workplace, addressing not only the architectural space, but also the general environmental setting and, in particular, the “training setting”, so as to develop the skills of employers and of employees.

In this framework, the concept of “training” is considered as an action that does not only consist in a transfer of skills, but that involves the whole person’s development, as a continuous learning process [10]. PATHWAYS findings highlighted that only a minor part of the worldwide existing training tools are evaluated and published in scientific literature, while a lot of strategies are often implemented in local settings by NGOs or in single companies and no scientific publication exist regarding them, therefore it is essential to broaden investigations.

In order to address this crucial issue for knowledge enhancement, the present study has been developed with the aim to identify and analyze the existing training tools for the management of sick employees in the workplace published in grey literature (i.e., on websites of Patients’ Associations, Institutional Organizations, Unions, Industrial Corporations, and Large Multinational Companies).

This mapping of existing training tools available in English on websites of Patients’ Associations, Institutional Organizations, Unions, Industrial Corporations, and Large Multinational Companies, provided to relevant stakeholders, in particular, to managers and employers, an overview of good practices and training programs in the workplace. This will allow for increasing the legislative, medical, psychological, and social knowledge for an effective management of employees with chronic diseases.

## 2. Materials and Methods

This study was carried out in the frame of the EU Joint Action “Chrodis Plus: Implementing good practices for chronic diseases” (http://chrodis.eu/), a three-year project that involves 42 beneficiaries that represent 20 European countries, that, between the various themes, covers the field of employment and chronic diseases.

### 2.1. Search Strategy

An exploratory overview of the online published materials was conducted through Google Scholar and Google, in order to identify the existing training tools for management of employee with NCDs that are currently used in EU countries and in three non-European countries: United States of America (USA), Canada, and Australia. Webpages of relevant European and International organizations, Disabled People’s Organizations (DPOs), NGOs, and scientific societies were also consulted (the detailed list of websites are included in Supplementary Materials).

An excel file was used in order to keep a record of the materials that were searched and to maintain a precise focus throughout the process. The excel file was used as a trail to document the search strategy and to include websites URLs, descriptions of the training tools used, and links to relevant documents.

Search terms included “chronic diseases”, “Non communicable diseases” or were related to specific diseases categories: “Cardiovascular diseases”, “Respiratory diseases”, “Metabolic syndromes”, “Mental disorders”, “Musculoskeletal disorders”, “Neurological disorders”, or specific diseases: “Diabetes”, “Multiple Sclerosis”, “Depression”, “Mental Health”, “Cancer”. Other search terms are related to the type of practices: “training tools”, “interventions”, “programs”, “Human Resources practices”, “policy”, “inclusion programs”, “work engagement”, and “return to work”.

### 2.2. Inclusion/Exclusion Criteria

Reports, guides, webinars, workshops, other resources produced by national ministries, political organizations, institutional organizations, patient’s associations, trade unions, world organizations, insurances, industrial corporations, and large multinational companies that were written in English, Italian, and Spanish and published between 2006 and 2018 were included. The decision to include publications in Spanish and Italian, beyond English, is related to the native language of the two authors who perform the search.

Documents were included if they reported an intervention, a program, HR practices, or policies to manage health issues or the RTW of employees with chronic conditions in the workplace. More specifically, the searched documents were only considered if the training tool included an intervention that was related to health or whether they presented a detailed description of organizational programs, practices, and policies to support a good re-integration of employees.

To have a more comprehensive overview of existing training tools for the management of the employees that are affected by chronic diseases in the labor market, it was decided to consider the training tools that were implemented by Large Multinational Companies, Patient’s Associations, Institutional Organizations, Insurances, Unions, and Industrial corporations. Training tools supporting the increase of legislative, medical, psychological, and social knowledge, both directly (e.g., specifically targeted at Chronic Diseases, CDs) and indirectly (e.g., CDs as parts of broader categories, disability or other), were considered.

Documents that include literature reviews, dissertations, and HR programs without effective implementation of the training tools were excluded instead.

## 3. Results

In total, 105 training tools to manage health issues, to facilitate inclusion, return, or retention to work of employees with chronic conditions in the workplace have been included. Table 1 shows the training tools that were considered for each category of chronic diseases.

The majority of these training programs for employers are focused on strategies of inclusion and RTW implemented by different categories of organizations (Table 2).

On the basis of material found, the training tools can be grouped in two main types: those that were implemented by Social and Institutional Organizations, external to companies (e.g., Patients’ Associations, Ministries, Industrial Corporations), and those implemented by Large Multinational Companies.

### 3.1. Training Tools Implemented by Social and Institutional Organizations

A key issue that emerged in the majority of documents found in this category, 90 out of 105, is that stigma, discrimination, stereotypes, fear, and lack of understanding are critical barriers for employees with PwCDs in the workplace.

Thus, these training tools mainly focus on those issues and can be classified in online training and/or face-to-face training for all employees (Table 3).

Online trainings are the most used in this category. They include guidelines, webinars, and factsheets, and they allow for disseminating political, legislative, medical, and psychological information to a greater number of people.

The majority of guidelines (*n* = 55) are primarily addressed to employers, managers, and Human Resources Staff (HRs) who are faced with challenges and opportunities that are implicated in the management of workers with CDs, in order to support them to stay at work or to assist them in the process of RTW after a period of sick leave.

Webinars are workshops that are transmitted online using video conferencing software’s, and their key feature is to be interactive instruments that allow the discussion on important matters in real-time. These trainings often explore how business processes can be improved for better productivity outcomes by employees with health problems.

Factsheets, Video, and Info graphic provide, instead, information regarding specific diseases, treatments, and their side effects, and outline practical suggestions for workplaces’ adaptation. They are mainly addressed to employers, employees suffering from health conditions, and PwCDs searching for a job.

“Workshop & Meeting” and “Course”, are instead tools that can be online or face-to-face training.

It is important to note that, of this typology, 50 training tools on 90 analyzed only contain generic information on how to improve the work environment. Therefore, there is a lack of information on how to implement these recommendations and customize the training.

### 3.2. Training Tools Implemented in Large Multinational Companies

The analyzed companies (Table 4) seem more interested in providing primary or secondary disease prevention through health prevention initiatives that are directed to all employees (e.g., through supplemental health insurance, family support services, healthy food in canteens, etc.) in order to improve their level of wellbeing and their quality of life, while the management of employees that are affected by CDs seems more difficult.

Wherever initiatives that are related to the management of employees affected by CDs exist, they are often included in the Diversity & Inclusion Policy of the Companies or carried out in collaboration with patients’ associations through, for example, specific workshops informing on the medical, psychological, and legislative aspects that are related to the targeted disease, both for employees and for their caregivers, online, or face-to-face.

Moreover, the companies often organize training initiatives to raise awareness among all employees on the topic of inclusion, against prejudices, stigma, conflicts, and marginalization.

An example of a good practice is described in Box 1.

Box 1 **Example of good practice (ENWHP, 2012).**

**“Strategic approach for sustaining people with chronic illnesses at work”**

**Delpeyrat, Saint Sever, France**

Three individual cases of people working with a chronic disease were analyzed, as well as the general situation regarding the health status of all the employees on the site. Specific communication means were developed in order to communicate largely on this project within the Delpeyrat Group.

This initiative had 3 main objectives:

- contribute to improving working conditions for all workers, whether they have a chronic pathology or not;
- maintain professional skills within the company;
- reduce absenteeism.

**Target group**

Management and HR; doctors; production and maintenance manager; H&S committee, healthcare professionals.

**Results**

**1. Facilitators**

- Strong willingness of the company management to build a specific approach on sustaining employees suffering from a chronic pathology at work.
- Structural organisation of a specific approach, based on both methodological material and the company culture.
- Elaboration of a dedicated communication strategy.

**2. Barriers**

- Wrong representations of both illness and/or activity.
- Invisibility of chronic pathologies at the workplace.
- Taboo aspect of the illness.
- Individual willingness of both management staff and workers suffering from a pathology

The companies usually support their sick employees to maintain job or RTW, mainly through work arrangements as flextime, compressed workweeks, telework/remote work, part-time, and job sharing.

A further result of our overview is related to the training of participants. The training tools found are addressed to all employees who work within the company, regardless of their role and without a specific categorization between different positions (e.g., employers, managers, coworkers, etc.). Such categorization could be useful in order to acquire the necessary knowledge for the management of employees that are affected by CDs, addressing the different needs of each category.

## 4. Discussion

The objective of this study was to identify and analyze the existing training tools directed to employers for the management of employees affected by CDs in Europe and in three non-European countries (USA, Canada, and Australia) that follow the occidental model.

Based on the organizations that created the training tools, the mapping of training programs revealed the existence of two different types of training tools: the first one includes strategies implemented by Social and Institutional Organizations (e.g., Patients’ Associations and Industrial Relations), the second one includes tools that were implemented in Large Multinational Companies.

In the first group, training tools are focused mainly on fighting stigma, discrimination, stereotypes, fear, and lack of inclusive and supportive workplaces, promoting accommodations and adjustments for workers with CDs. The effectiveness of these trainings seems to be affected by the work environment, the work tasks, and by the relationship between co-workers and managers [11,12,13,14].

This type of training tools is, in general, implemented in an online modality. Online trainings have a lot of advantages, especially for employers, providing them with the skills that they need in their working environment, as well as boosting their qualifications in the workplace.

This kind of training does not allow a face-to-face interaction and it creates one-way communication, without mutual exchange that would allow to feel themselves active actors in the learning process.

In the second group, the training tools implemented in the Large Multinational Companies are instead mainly focused on primary or secondary disease prevention that aims to reduce the risks on health status. Scientific literature proposes many definitions of the prevention [15,16]. The Primary prevention realizes interventions in which the central objective is to improve the health status of the whole population, without specific attention towards individual risks. Secondary and tertiary prevention interventions are addressed to persons that already have some health conditions in order to reduce or reverse the negative consequences of a disease in a workplace [17]. These initiatives propose a more individual approach (e.g., providing training sessions addressed to groups of employees in order to decrease the physical workload or empowering sessions to increase the work control).

In general, these programs do not consider the problem of presenteeism of ill employees, which occurs “when an employee goes to work despite a medical illness that will prevent him or her from fully functioning at work” [18]. It represents a complex issue for employers, because it would require some knowledge of a potentially wide range of illnesses [8]. According to Schultz et al. [19], an integrated approach to mitigate the effects of presenteeism might include, in training programs, topics, such as disability management, disease management, behavioral health management, and absence management.

Even if Large Multinational Companies do not directly deal with presenteeism, it is important to note that they tend to use flexible work arrangements as a mean to decrease the presenteeism and to manage the (re)integration to work of PwCDs.

Flexible work arrangements enable employers and employees to decide when (e.g., flextime), where (e.g., working from home), and for how long (e.g., sabbaticals) they should engage in work-related tasks [20]. It is shown that flexible work arrangements reduce sickness absence, turnover, overtime, stress, and improvements in recruitment and productivity [7]. However, even if these improvements have the potential of making a monetary contribution, they do not influence the soft skills of employers in managing employees that are affected by CDs.

In addition, our study highlights that Large Multinational Companies often use coaching sessions that aimed to improve employers’ interpersonal skills and management abilities, but these trainings do not usually include information regarding the legal, medical, and psychological aspects, which would be useful for an adequate management of employees with CDs.

It is important to note that the training tools of both categories are usually structured so as to provide generic recommendations, which aim to help employers to improve the work environment in relation to access, return, and maintenance to the work of employees with disability, but they lack of specific information on how to implement these actions in concrete terms, customizing—if needed—the training on the base of the diseases of the employees of the company.

Finally, some limitations should be mentioned. First, even though our search was extensive, we are aware that some material was not found due to the databases used (only Google Scholar and Google) or was in other languages. Our search, in fact, mainly captured the more standardized training tools, usually developed by big companies or organizations, which publish their training tools on the website, but we cannot exclude that other training tools, developed at local level, exist. Secondly, given the great heterogeneity of the training tools identified, in the analysis it was not possible to take into consideration all of the contextual features, such as the dimensions of the organization or the country where the training tools were carried out.

Despite these limitations, the study also presents strengths. First, it searched for materials on training tools directly from the websites of the organizations who created them; in this way, it was possible to also find those training tools that are not published in scientific literature. Second, it presents 105 training programs and interventions that were developed for employers and employees, conducted with different methodologies; this allows for increasing the knowledge for the effective management of employees with chronic diseases, giving information to key stakeholders to create new programs or to adapt the existing ones.

In order to have a complete framework of the issue, it could be interesting to explore the field of vocational rehabilitation. There is, in fact, evidence that rehabilitation with a multidisciplinary focus on work-related demands effectively improves work ability and quickens the return to work in patients with chronic disorders [21,22,23]. A joint work on the patients, through vocational rehabilitation, and on the workplace, through training tools for employers, may in fact be crucial. Improved aftercare treatment, in fact, requires a focus on employer participation and involvement within the actual work environment; on the other hand, effective training that is addressed to employers can benefit from the key role of vocational rehabilitation in a good return to work process.

## 5. Conclusions

To a large extent, the training programs that were identified in our search are mainly focused on the creation of inclusive workplaces, promoting accommodations and adjustments for workers with disability, by mean of generic recommendations that suggest to the employers how to develop a better management of ill employees, but information on what concrete actions have to be implemented are often missing. Moreover, there is a need to develop training tools that are also accessible also employers of small enterprises that usually have limited economic resources and that are not a target of specific training tools.

Therefore, further research is necessary in order to better explore the contextual characteristics that may hinder or facilitate the implementation of training tools to create an inclusive work environment.

## Figures and Tables

**Table 1:** Number of training tools considered for each chronic diseases.

Illness	*n*
Cancer	25
Multiple Sclerosis	4
Rheumatic Diseases	2
Mental Health	11
Diabetes	2
Cardiovascular Diseases	2
Allergy and Airways Diseases	1
Disability in general *	58

* Training tools not focused on a specific diseases/diagnosis, but that target “people with a disability”, not better specified.

**Table 2:** Number of training tool found for each category of organization.

Organizations	Training Tool (*n*)
European Patients’ Associations	30
European Institutional Organizations	22
Large Multinational Companies	15
European Industrial Relations	4
Canadian Institutional Organizations	4
Canadian Patients’ Associations	4
American Institutional Organizations	9
American Patients’ Associations	5
Australian Institutional Organizations	3
Australian Patients’ Associations	4
World Associations	5

**Table 3:** Classification of training tools.

N	Organization	Program’s Name	Type of Tool Used	Chronic Disease Considered
1	ENWHP—The European Network for Workplace Health Promotion	PH WORK: Promoting Healthy Work for people with chronic illness	Guidelines online	Disability in general
2	The Great-West Life Centre for Mental Health in the Workplace	Supporting Employee Success	Guidelines online	Mental Health
3	EPF—European Patients Forum	Recommendations to promote better inclusion of people with chronic conditions in the workplace in the context of the European Pillar of Social Rights	Leaflet and Recommendations	Disability in general
4	The Academic Network of European Disability Experts (ANED)	DOTCOM: the Disability Online Tool of the Commission	Web links	Disability in general
5	European Multiple Sclerosis Platform (EMSP)	A Practical Toolkit for Employers	Guidelines online	Multiple Sclerosis
6	European Public Health Alliance	Mental Health Europe (MHE)	Infographic and video	Mental Health
7	IDF Europe—International Diabetes Federation	The European Men’s Health Forum (EMHF)	Guidelines and video	Diabetes
8	ECDC—European Centre for Disease Prevention and Control	Early Intervention	Guidelines online	Disability in general
9	FESCA—Federation of European Scleroderma Associations	Fit For Work	Guidelines online	Scleroderma
10	INCa—The French National Cancer Institute	Cancer Control Plan	Guidelines online	Cancer
11	INCa—The French National Cancer Institute	A Business Club	Guidelines online	Cancer
12	EFNA—European Federation of Neurological Associations	#MAKEWORKWORK	Guidelines online	Mental Health
13	EDF—European Disability Forum	Disability Advocates Project	Guidelines online	Disability in general
14	IDA—International Disability Alliance	BRIDGE CRPD-SDG Training	Workshop and meeting	Disability in general
15	ECPC—European Cancer Patient Coalition	Cancer Advocacy Academy	Guidelines online	Cancer
16	EFA—European Federation of Allergy and Airways Diseases Patients’ Association	Meet and Greet EU Institutions	Workshop and meeting	Allergy and Airways Diseases
17	EULAR—European League Against Rheumatism	EULAR COURSE	Workshop and meeting	Rheumatic Diseases
18	EORTC—European Organisation for Research and Treatment of Cancer	Course dedicated to Patients and Caregivers interested in Cancer Clinical Research	Workshop and meeting	Cancer
19	EPA—European Psychiatric Association	EPA COURSES: Congress EPA Course, EPA Itinerant Course, Faculty Workshop	Workshop and meeting	Mental Health
20	Mental Health Europe	How to promote mental health in the workplace?	infographic	Mental Health
21	Ministry of Social Affairs—Austria	fit2work	Guidelines online	Disability in general
22	Graz City Council.	In-house Crisis Prevention and Crisis Intervention	Guidelines online	Disability in general
23	OÖGKK (Upper Austrian Sickness Funds), Austrian employers insurance	Social Coaching	Guidelines online	Disability in general
24	Jobcentrum	Rentree—working after cancer	Workshop and meeting	Cancer
25	Jobcentrum	Stand up against work by cancer	Workshop and meeting	Cancer
26	The Big C—Coffee for Cancer	The Big C.Hallenge—Coffee & Chances	Workshop and meeting	Cancer
27	Ministry of Labour and Social Policy, Employment Agency—Bulgaria	National Programme for employment and training of people with disabilities	Workshop and meeting	Disability in general
28	Government Denmark	Danish return-to-work project	Workshop and meeting	Disability in general
29	Huset Venture	The House of Venture	Workshop and meeting	Disability in general
30	The Ministry of Social Affairs and Health—Finland	Career opportunities for people with partial work ability—Training for work ability coordinators	Workshop and meeting	Disability in general
31	AISM—Associazione Italiana Sclerosi Multipla—Italy	Work: national collective agreements and multiple sclerosis	Guidelines online	Multiple Sclerosis
32	AISLA—Associazione Italiana Sclerosi Laterale Amiotrofica—Italy	to care not to care	Guidelines online	Multiple Sclerosis
33	Europa Donna—Italy	case of essential news	Guidelines online	Cancer
34	Europa Donna—Italy	collaborations and activities with companies	Guidelines online and workshop	Cancer
35	Europa Donna—Italy	Breast cancer: the relationship between the worker and the post-disease return	Guidelines online and workshop	Cancer
36	AIMAC—Associazione Italiane Malati di Cancro—Italy	Rights of the patients	Guidelines online	Cancer
37	AIMAC—Associazione Italiane Malati di Cancro—Italy	Workers with cancer: 10 tips to the employer	Guidelines online	Cancer
38	FAVO—Federazione delle Associazioni di Volontariato in Oncologia—Italy	Patient’s right	Guidelines online	Cancer
39	APMAR—Associazione Nazionale Persone con Malattie Reumatologiche e Rare—Italy	Focus on diseases	Guidelines online	Rheumatic Diseases
40	AIL Onlus—Associazione Italiana contro le Leucemie-linfomi e myeloma—Italy	Rights and benefits	Guidelines online	Cancer
41	IncontraDonna Onlus—Italy	Corporate Welfare	Guidelines online	Cancer
42	Fondazione CIGNO Onlus—Italy	Meet Woman	Guidelines online	Cancer
43	ANDOS Onlus—Associazione Nazionale Donne Operate al Seno—Italy	AL LAVORO! PERMESSI E CONGEDI	Guidelines online	Cancer
44	DuPont Sustainable Solutions	STOP^®^ Safety Training Observation Program Overview	Meeting, video, guidelines	Disability in general
45	Employment and Training Corporation	Supported and Sheltered Employment Training for Persons with Disability Project	Workshop and meeting	Disability in general
46	Employment and Training Corporation	OPII Empowering People for More Jobs and a Better Quality of Life ESF	Workshop and meeting	Disability in general
47	CEAC (European private training group) and Fomento del Trabajo Nacional (Catalonia)	GESTPYME	Workshop and Guidelines	Disability in general
48	ISTAS (autonomous technical foundation) supported by ‘Confederación Sindical de Comisiones Obreras’	’Por experiencia’	Video, workshop	Disability in general
49	ASTRA AB	ASTRA	Dossier	Disability in general
50	Lloyds TSB	Positive about disability	Course	Disability in general
51	EESC—European Economic and Social Committee	Permanent study group on disability rights	Forum	Disability in general
52	The BC Centre for Employment Excellence	Workplace Check-up	Guidelines online	Disability in general
53	ACAS (Advisory, Conciliation and Arbitration Service)	Disability discrimination: key points for the workplace	Guidelines online	Disability in general
54	The Australian Prevention Partnership Centre	Prevention Landscape: The status of prevention programs in Australian states and territories following two national prevention initiatives	Guidelines online	Disability in general
55	The Royal Australian College of General Practitioners (RACGP)	Improving chronic disease management in your general practice	Webinar	Disability in general
56	Cancer Council—Australia	Cancer, work and you	Fact sheets	Cancer
57	National Heart Foundation of Australia	Active workplaces	Guidelines online	Disability in general
58	Australian Human rights commission	Good practice good business factsheets	Guidelines online	Chronic Disease in general
59	MSWA: Supporting People With All Neurological Conditions	Employer resources	Guidelines online	Mental Health
60	Mentally Healthy Workplace Alliance	National Workplace Program	Guidelines online	Mental Health
61	Workplace Health in Australia	Best-Practice Guidelines Workplace Health in Australia	Guidelines online	Disability in general
62	CDC—Centers for Disease Control and Prevention	Work @ Health Program	Guidelines online	Disability in general
63	EARN (Employer Assistance and Resource Network on Disability Inclusion)	Training center	Webinar	Disability in general
64	Utah Department of Health	Affordable Care Act and Worksites	Guidelines online	Disability in general
65	National Association of Chronic Disease Directors—USA	Workplace Assessment	Guidelines online	Disability in general
66	Bender Consulting Services	Disability Employment Strategy and Training	Guidelines online	Disability in general
67	Health Matters	Browse our general workplace training course portfolio	Course	Disability in general
68	Workplace Safety & Prevention Services (WSPS)	Workplace Mental Health	workshop online	Mental Health
69	Mental Health America (MHA)	Workplace Wellness	Guidelines online	Mental Health
70	The Office of Disability Employment Policy (ODEP)	Disability Employment Policy Resources	Guidelines online	Disability in general
71	American Cancer Society	Americans With Disabilities Act: Information for People Facing Cancer	Guidelines online	Cancer
72	Northeast Business Group on Health (NEBGH)	Cancer and the Workplace: The Employer Perspective	Guidelines online	Cancer
73	The American Heart Association	The American Heart Association’s Workplace Health Solutions	Guidelines online	Cardiovascular Diseases
74	American Diabetes Association	Stop Diabetes @ Work	Guidelines online	Diabetes
75	National Multiple sclerosis society	Employment Matters: Managing MS in the Workplace	Guidelines online, video	Multiple Sclerosis
76	EBC—European Brain Council	Not My Self Today	Guidelines online, video, workshop	Mental Health
77	The Great-West Life Centre for Mental Health in the Workplace	Supporting Employee Supporting Employee Success	Video	Disability in general
78	The Great-West Life Centre for Mental Health in the Workplace	Management Training	Video	Disability in general
79	BC Alliance for Healthy Living	Working on Wellness in Strategic Populations (WoW)	Guidelines online	Disability in general
80	Canadian Cardiovascular Society	Trainee Program at CCC & Networking Lunch	Workshop	Cardiovascular Diseases
81	The Chronic Disease Prevention Alliance of Canada (CDPAC)	Webinars	Webinars	Disability in general
82	Canadian Public Health Association	Planning and assessment tool for chronic disease prevention and management	Guidelines online	Disability in general
83	SMRC—Self-management resource center	Workplace Chronic Disease Self-Managment	Guidelines and Workshop	Disability in general
84	ILO Global Business and Disability Network	ILO Global Business and Disability Network	Guidelines online	Disability in general
85	WHO chronic diseaes and health promotion	Stop the global epidemic of chronic disease: A practical guide to successful advocacy	Guidelines online	Disability in general
86	IPOS—International Psycho-Oncology Society	For professional: course/ training	Course	Cancer
87	IPOS—International Psycho-Oncology Society	Psychosocial Academy	Course	Cancer
88	Organisation for Economic Co-operation and Development (OCSE)	Health Workforce Policies in OECD Countries	Guidelines online	Disability in general
89	The world bank	Out of the Shadows: Making Mental Health a Global Priority	Workshop	Mental Health
90	Forsikring & Pension—Danimark	Hold nu fast!—en guide til at fa sygemeldte tilbage i job	Guidelines online	Disability in general

**Table 4:** Training tools implemented by Large Multinational Companies.

N	Organization	Program’s Name	Type of Tool Used	Chronic Disease Considered
1	Delpeyrat	Strategic approach for sustaining people with chronic illnesses at work	Guidelines online	Disability in general
2	ARACT Aquitaine	Implementation of a website dedicated to work and chronic illness	Guidelines online	Disability in general
3	Ford	Ford Werke—Disability Management	Workshop, training individual	Disability in general
4	HELLAS EAP	HELLAS Employee assistance programs	Guidelines online	Disability in general
5	ABBOTT	“Employment programme for graduates with disabilities & Occupational Health programme	Leaflets, poster, workshop	Disability in general
6	EMA	EMA—AHEAD and cancer	Leaflets, poster, workshop	Cancer
7	IKEA	PRO JOB—Lavorare durante e dopo il cancro: una risorsa per l’impresa e per i lavoratori	Guidelines online, workshop	Cancer
8	Eni	PRO JOB—Lavorare durante e dopo il cancro: una risorsa per l’impresa e per i lavoratori	Guidelines online, workshop	Cancer
9	Generali	WELL-BEING	Workshop	Disability in general
10	Enel	Diversity and inclusion: the key to our success Policy on Diversity and Inclusion;	Course	Disability in general
11	Telecom	Managing Diversity	Guidelines online, workshop	Disability in general
12	Sodexo	Diversity and Inclusion: how we act	Workshop	Disability in general
13	Pirelli	Welfare and initiatives for the internal community	Workshop	Disability in general
14	Deutsche Bank	Health and work-life balance	Course	Disability in general
15	Credit Suisse	Flexibility & Health	Workshop	Disability in general

## Supplementary Materials

The following are available online at https://www.mdpi.com/1660-4601/16/5/718/s1, S1: Online sources consulted for the study.
