# Supplementary material for: Chronic Diseases & Employment: An Overview of Existing Training Tools for Employers
Source: Int J Environ Res Public Health. 2019 Feb 28;16(5):718. doi: 10.3390/ijerph16050718 (PMC6427204; doi:10.3390/ijerph16050718)
Supplement: Supplementary file 1 [file ijerph-16-00718-s001.pdf]

Supplementary Materials

Online sources consulted for the study

Report and Guidelines

1. CEAC and Fomento del Trabajo Nacional. *GESTPYME*, ENWHP: Spain, n.d.; Available online: http://www.enwhp.org/fileadmin/downloads/models/SME-Project/Spain/GESTPYME.pdf (accessed not declare)
2. Confederación Sindical de Comisiones Obreras. *ISTAS*, ENWHP: Spain, n.d.; available online: http://www.enwhp.org/fileadmin/downloads/models/SME-Project/Spain/ISTAS.pdf (accessed not declare)
3. DuPont Sustainable Solutions. *STOP® Safety Training Observation Program Overview*, USA, 2016; Available online: http://www.training.dupont.com/content/pdf/dupont-stop/stop-overview_factsheet.pdf (accessed not declare).
4. European Multiple Sclerosis Platform (EMSP). *A Practical Toolkit for Employers*, EMSP: Brussels, Belgium, n.d.; Available online: http://www.emsp.org/wp-content/uploads/2016/01/EMSP_PPP-Toolkit_20ppA4_email_v2.pdf (accessed not declare)
5. European Network for Workplace Health Promotion (ENWHP). *Promoting healthy work for workers with chronic illness: A guide to good practice*, ENWHP: Spain, 2012; Available online: http://www.enwhp.org/uploads/media/ENWHP_Guide_PH_Work_final.pdf (accessed not declare)
6. European Network for Workplace Health Promotion (ENWHP). *Social Coaching*, ENWHP: Spain, 2012; available online :http://www.enwhp.org/fileadmin/user_upload/pdf/PH_Work_Social_Coaching_Austria.pdf (accessed not declare)
7. European Network for Workplace Health Promotion (ENWHP). *The socio-economic enterprise Huset Venture*, ENWHP: Spain, 2012; Available online: http://www.enwhp.org/fileadmin/user_upload/pdf/PH_Work_Huset_Venture_Denmark.pdf , (accessed not declare).
8. European Network for Workplace Health Promotion (ENWHP). *Strategic approach for sustaining people with chronic illnesses at work"*, ENWHP: Spain, 2012; Available online: http://www.enwhp.org/fileadmin/user_upload/pdf/PH_Work_Delpeyrat_France.pdf (accessed not declare)
9. European Network for Workplace Health Promotion (ENWHP). *Implementation of a website dedicated to work and chronic illness*, ENWHP: Spain, 2012; Available online: http://www.enwhp.org/fileadmin/user_upload/pdf/PH_Work_website_work___chronic_illness_France.pdf (accessed not declare)
10. European Network for Workplace Health Promotion (ENWHP). *Ford Werke – Disability Management*, ENWHP: Spain, 2012; Available online: http://www.enwhp.org/fileadmin/user_upload/pdf/PH_Work_Disability_Management_Ford_Germany.pdf (accessed not declare)
11. European Network for Workplace Health Promotion (ENWHP). *HELLAS EAP*, ENWHP: Spain, 2012; Available online: http://www.enwhp.org/fileadmin/user_upload/pdf/PH_Work_Hellas_EAP_Greece.pdf (accessed not declare)
12. European Network for Workplace Health Promotion (ENWHP). *Abbott*, ENWHP: Spain, 2012; Available online: http://www.enwhp.org/fileadmin/user_upload/pdf/PH_Work_Abbott_Ireland.pdf (accessed not declare)
13. European Patients' Forum . *Recommendations to promote better inclusion of people with chronic conditions in the workplace in the context of the European Pillar of Social Rights*, EPF: Belgium, 2012; Available online:

http://www.eu-patient.eu/globalassets/policy/employment/recommendations-for-policy-makers.pdf (accessed not declare)
14. Government Denmark. *The Danish National Return-to-work programme: Denmark*, ENWHP: Denmark, 2015; Available online: https://osha.europa.eu/en/tools-and-publications/publications/denmark-danish-national-return-work-programme/view (accessed 18 December 2015)
15. The Ministry of Social Affairs and Health – Finland. *Career opportunities for people with partial work ability*, ENWHP: Finland, 2012; Available online: https://stm.fi/documents/1271139/5417419/Career+Opportunities+for+Persons+With+Partial+Work+Ability+2017.pdf/9766f671-a798-4b74-8f38-1f93b05ff4f6 (accessed not declare)
16. MSWA. *Guide for employers*, MSWA: Australia, 2016; Available online: https://mswa.org.au/assets/stories/MSA_GuideForEmployers.pdf (accessed not declare)
17. Northeast Business Group on Health (NEBGH). *Cancer and the Workplace: The Employer, Perspective*, NEBGH: New York, 2015; Available online http://nebgh.org/wp-content/uploads/2015/10/CancerWorkplace_FINAL.pdf (accessed not declare)
18. The Health & Productivity Institute of Australia (HAPIA). *Best-Practice Guidelines*, HAPIA: Australia, 2015 Available online: http://www.workplacehealth.org.au/UnderstandWorkplaceHealth/best-practice-guidlines (accessed not declare)

Websites

1. **The Academic Network of European Disability Experts (ANED)**
https://www.disability-europe.net/dotcom
2. **ACAS (Advisory, Conciliation and Arbitration Service)**
http://www.acas.org.uk/index.aspx?articleid=1859
3. **AIMAC - Associazione Italiane Malati di Cancro**
https://www.aimac.it/diritti-del-malato
4. **AISLA - Associazione Italiana Sclerosi Laterale Amiotrofica- Italy**
http://www.aisla.it/formazione/
5. **AISM - Associazione Italiana Sclerosi Multipla- Italy**
https://www.aism.it/index.aspx?codpage=sclerosi_multipla_contratti_lavoro
6. **American Diabetes Association**
http://www.diabetes.org/in-my-community/awareness-programs/stop-diabetes-at-work/
7. **ANDOS Onlus - Associazione Nazionale Donne Operate al Seno - Italy**
http://www.andosonlusnazionale.it/informazioni-utili/al-lavoro.html
8. **Australian Human Rights Commission**
https://www.humanrights.gov.au/employers/good-practice-good-business-factsheets
9. **The American Heart Association**
https://www.heart.org/en/professional/workplace-health
10. **APMAR - Associazione Nazionale Persone con Malattie Reumatologiche e Rare- Italy**
https://www.apmar.it/
11. **The Australian Prevention Partnership Centre**
https://preventioncentre.org.au/
12. **The Big C - Coffee for Cancer**
https://www.thebigc-hallenge.com/
13. **Bender Consulting Services**
https://www.benderconsult.com/our%20services/disability-employment-strategy-and-training
14. **The BC Centre for Employment Excellence**
http://www.cfeebc.org/resource/workplace-check-up/
15. **Cancer Council - Australia**

https://www.cancercouncil.com.au/cancer-information/work-and-cancer/for-workplaces-employers/?pp=110587
16. **Canadian Public Health Association**
http://www.nccmt.ca/knowledge-repositories/search/81
17. **CDC - Centers for Disease Control and Prevention**
https://www.cdc.gov/workplacehealthpromotion/initiatives/workathealth/index.html
18. **The Chronic Disease Prevention Alliance of Canada (CDPAC)**
http://www.cdpac.ca/content.php?doc=180
19. **Credit Suisse - Flexibility & Health**
https://www.credit-suisse.com/corporate/en/responsibility/employer/flexibility-health.html
20. **Deutsche Bank - Health and work-life balance**
https://www.db.com/cr/en/our-people/health--and--work-life--balance.html
21. **EARN (Employer Assistance and Resource Network on Disability Inclusion)**
http://www.askearn.org/earn-training-center/
22. **EBC- European Brain Council**
http://www.notmyselftoday.ca/why-join/
23. **ECDC - European Centre for Disease Prevention and Control**
https://ecdc.europa.eu/en/home
24. **ECPC - European Cancer Patient Coalition**
http://www.ecpc.org/
25. **EDF - European Disability Forum**
http://www.edf-feph.org/disability-advocates-project
26. **EFA - European Federation of Allergy and Airways Diseases Patients' Association**
http://www.efanet.org/what-we-do/capacity-building
27. **EFNA - European Federation of Neurological Associations**
http://www.brainmindpain.eu/written-declaration-access-to-employment/
28. **Enel - Diversity and inclusion: the key to our success Policy on Diversity and Inclusion**
https://www.enel.com/careers/explore-enel/diversity-inclusion
29. **Employment and Training Corporation - Malta**
https://investinginyourfuture.gov.mt/project/equal-opportunities-life-long-learning-and-employment/employment-support-for-persons-with-disabilities-37060626
30. **EORTC - European Organisation for Research and Treatment of Cancer**
http://www.eortc.org/education-training/
31. **EPA - European Psychiatric Association**
http://www.europsy.net/education/courses/itinerant-courses/
32. **EULAR - European League Against Rheumatism**
https://www.eular.org/
33. **Europa Donna - Italy**
http://europadonna.it/tumore-e-lavoro/
34. **European Public Health Alliance**
https://mhe-sme.org/
35. **FAVO - Federazione delle Associazioni di Volontariato in Oncologia - Italy**
https://www.favo.it/diritti-malato-normativa.html
36. **FESCA, Federation of European Scleroderma Associations**
http://www.worldsclerofound.org/fesca/
37. **Generali – Wellbeing**
https://www.generali.com/it/our-responsibilities/investing-in-our-people/health-and-safety
38. **The Great-West Life Centre for Mental Health in the Workplace**
https://www.workplacestrategiesformentalhealth.com/managing-workplace-issues/supporting-employee-success-a-tool-to-plan-accommodations

39. **IDF Europe - International Diabetes Federation**
https://www.idf.org/our-network/regions-members/europe/welcome.html
40. **IDA - International Disability Alliance**
http://www.internationaldisabilityalliance.org/content/bridge-crpd-sdg-training
41. **INCa - The French National Cancer Institute**
http://en.e-cancer.fr/The-Cancer-Plan-2014-2019
42. **ILO Global Business and Disability Network**
http://www.businessanddisability.org/satools/?mod=about
43. **IncontraDonna Onlus - Italy**
http://www.incontradonna.it/index/eventi-e-progetti/582-il-welfare-aziendale-funziona-e-incontradonna-lo-sostiene-seminari-sulla-salute-della-donna-e-dell-uomo-per-i-dipendenti-di-ferrovie-dello-stato
44. **Mental Health Europe**
https://mhe-sme.org/
45. **Mentally Healthy Workplace Alliance**
https://www.headsup.org.au/training-and-resources/educational-and-training/national-workplace-program
46. **Mental Health America (MHA)**
http://www.mentalhealthamerica.net/workplace-wellness#Top5Job
47. **Ministry of Labour and Social Policy, Employment Agency**
https://egov.bg/wps/portal/en
48. **National Heart Foundation of Australia**
https://www.heartfoundation.org.au/for-professionals/physical-activity/workplace-wellness
49. **The Office of Disability Employment Policy (ODEP)**
https://www.dol.gov/odep/topics/Mental_Health.html
50. **Pirelli - Welfare and initiatives for the internal community**
http://annual-report-2014.pirelli.it/management-report/en/report-value-chain-responsible-management-0/social-dimension/internal-community/welfare-and#.Wr4vgC5uYdU
51. **Sodexo – Diversity and Inclusion: how we act**
https://www.sodexo.com/home/corporate-responsibility/diversity-and-inclusion/how-we-act.html
52. **Jobcentrum**
https://www.rentree.eu/
53. **The World Bank**
http://www.worldbank.org/en/events/2016/03/09/out-of-the-shadows-making-mental-health-a-global-priority
